# μPADs for a Safer Night’s Sleep: An Alternative for Quality Control of Melatonin Pharmaceutical Formulations

**DOI:** 10.1021/acsomega.6c03837

**Published:** 2026-08-14

**Authors:** Ana Julia Aparecida Ramos, Laís Canniatti Brazaca, Eduardo Luiz Rossini, Manoel de Jesus de Aquino Lima, Emanuel Carrilho

**Affiliations:** † Instituto de Química de São Carlos, 28133 Universidade de São Paulo, 13566-590 São Carlos, SP, Brazil; ‡ Instituto Nacional de Ciência e Tecnologia de Bioanalítica − Lauro Kubota (INCTBio-LK), 13083-970 Campinas, SP, Brazil

## Abstract

Melatonin, known as the “sleep hormone,” has its primary function in regulating the circadian cycle, a 24 h sleep–wake pattern that leads to significant physical, mental, and behavioral changes. The use of melatonin in pharmaceutical formulations gained popularity as a tool to combat sleep disorders such as insomnia and has been increasing in the last few decades. Melatonin-based pharmaceutical formulations are classified as dietary supplements, which leads to a different perspective on the necessity of strict quality control for these products. There are already extensive descriptions in the literature of the serious side effects associated with the administration of those formulations, including, for example, higher dosages than indicated on the label or contamination by other hormones. Still, there is a gap in devices that could make an easy and low-cost determination of melatonin. This work presents a paper-based sensor for the quality control of melatonin in pharmaceutical formulations, utilizing the modified Van Urk reaction. This reaction causes a color change from white to blue on the paper surface, correlating with the melatonin concentration. The constructed sensor performed well in quantifying melatonin in solid (0.22 mg/tablet) and liquid (0.20 mg/drop) pharmaceutical formulations, considering the maximum standard deviation when compared with results obtained by a gold-standard technique (liquid chromatography). The developed method demonstrated good linearity (R^2^ = 0.9915) over a melatonin concentration range of 40–300 mg L^–1^ and exhibited a specific response to the hormone when tested against the vehicles and excipients of the pharmaceutical formulations. Therefore, the proposed sensor demonstrates excellent potential as an inexpensive tool for quality control, introducing an innovative approach to the use of paper-based microfluidic devices in this field.

## Graphical abstract

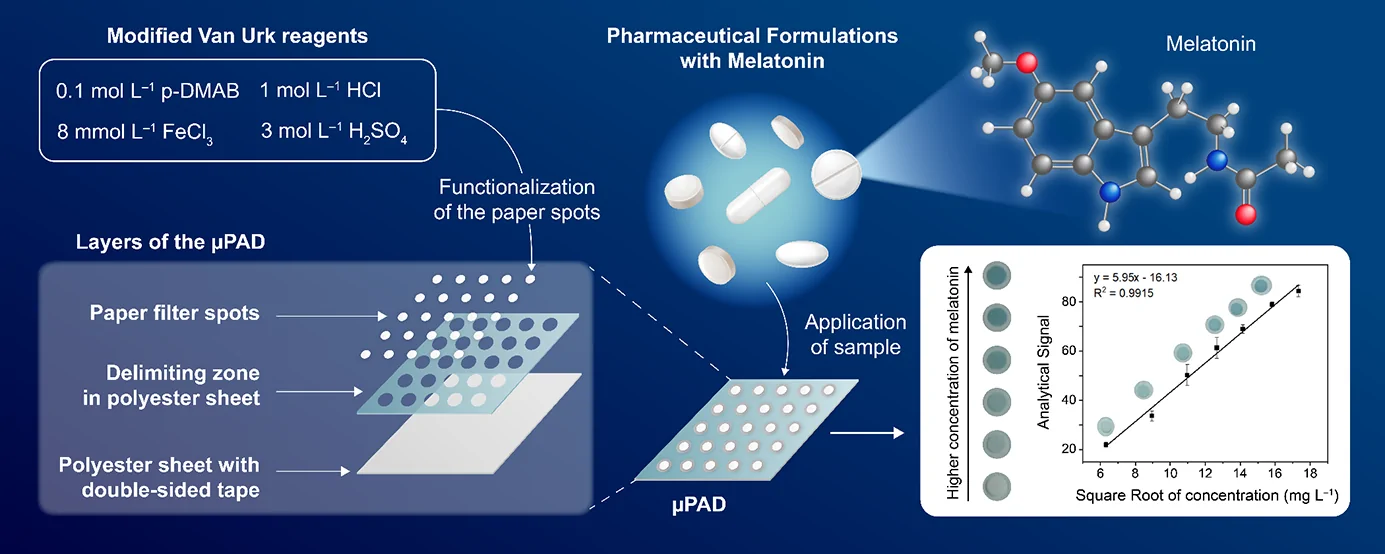

## 1 Introduction

Melatonin (N-acetyl 5-methoxy tryptamine) is one of the most important hormones regulating the circadian rhythm. This 24 h cycle reflects behavior and physical and mental changes in any living being.^1^ The hormone is mainly produced in the pineal gland. When it reaches the blood, reflections in the human body commonly associated with the sleep stage occur, such as a decrease in body temperature and blood pressure.^2^ Because of these effects, melatonin has great potential for treating sleep disorders like insomnia.^3^ Melatonin is also being studied for its potential role in other health-related processes, such as immunity and aging, as well as for its potential to prevent diseases like Alzheimer’s and certain types of cancer.^4–6^

The current popularity of melatonin as a sleep-promoting drug is continually rising, with its consumption increasing by 0.4% between 1999 and 2000 and by 2.1% between 2017 and 2018.^7^ However, the FDA (Food and Drug Administration) does not label melatonin as a drug, but as a dietary supplement. In this scenario, there is a lack of precise guidelines on how to consume it safely, including dosage, labeling, and other relevant considerations.^8^ In Brazil, a similar layout takes place: ANVISA, the federal regulatory agency in sanitary regulation, approved the consumption of 0.21 mg of melatonin per day for adults (>19 years old) with sleep issues, but it is possible to obtain pharmaceutical formulations with higher quantities of the hormone if those are labeled as a food supplement.^9^ Due to the lack of a standardized quality control, concerns have been raised about the quality and safety of commercially available melatonin products. Previous studies have described that the melatonin content in pharmaceutical formulations in the USA varies from −83% to 487% of the labeled amount, potentially promoting side effects such as headaches, nausea, and others.^10,11^

Several methods for determining melatonin in pharmaceutical formulations have been described, utilizing chromatography, UV–vis spectroscopy, or fluorescence spectroscopy techniques.^12,13^ Although these techniques can provide precise and reliable results, they also present disadvantages such as high cost, the need for specialized operators, being time-consuming, and more. As of September 2024, ANVISA has mandated that the notification of dietary supplements will be compulsory. This regulatory requirement will include a stability study of the formulation to ensure that the supplement consistently provides the labeled active ingredient throughout its shelf life. The principal challenge to meeting this health regulation is the significant financial burden associated with this process.^14^ Therefore, developing low-cost but equally effective methods could benefit the pharmaceutical formulation quality control of melatonin.

Microfluidic paper-based analytical devices (μPADs) are valuable tools for analytical applications in various areas, thanks to the numerous advantages of paper (e.g., low cost, flexibility, high surface area, and the ability to adsorb small sample and reagent volumes in the microfluidic channels).^15^ Many examples of μPADs play a key role in quality analysis for food safety. These include, for example, devices for detecting targets such as the bacteria *Escherichia coli* in milk^16^ or the pesticide carbofuran in rice.^17^ However, to our knowledge, there are no records of the use of μPADs for quality control in the field of medication and food supplement safety.

In this study, we developed a paper-based microfluidic sensor to detect melatonin in pharmaceutical formulations, providing an innovative tool for quality control. The device is based on a colorimetric reaction using the “Van Urk” reagent, which produces a purple-blue color in the presence of indoles.^11^ The developed sensor quantifies and detects melatonin in pharmaceutical formulations using smartphones for data acquisition and processing. To our knowledge, this is the first μPAD for determining melatonin in pharmaceutical formulations.

We demonstrated the effectiveness of the sensor by comparing it with the gold standard analytical technique of high-efficiency liquid chromatography (HPLC) using solid and liquid pharmaceutical samples. The constructed sensor presented a relative error of −8% for solid samples and 12% for liquid samples when compared to HPLC, demonstrating great potential for the sensor to serve as a low-cost, accessible, and easy-to-use screening device for the quality control of food supplements containing melatonin.

## 2 Experimental Section

### 2.1 Design and Fabrication of the Sensor

The sensor was created in three steps: constructing paper spots, delimiting their area, and assembling the device with a polyester sheet and double-sided tape. We used a CO_2_ laser cutter to cut 7 mm diameter spots in filter paper and slightly larger spots (11 mm in diameter) in a polyester sheet to prevent reagent leaks as the base. We assembled these two parts, attaching them to a transparent polyester sheet using double-sided tape to introduce the design presented in Figure 1A (Full information is provided in the Supporting Information, S1). The average cost of the materials used in this approach is $0.009 per spot.

**Figure 1:**
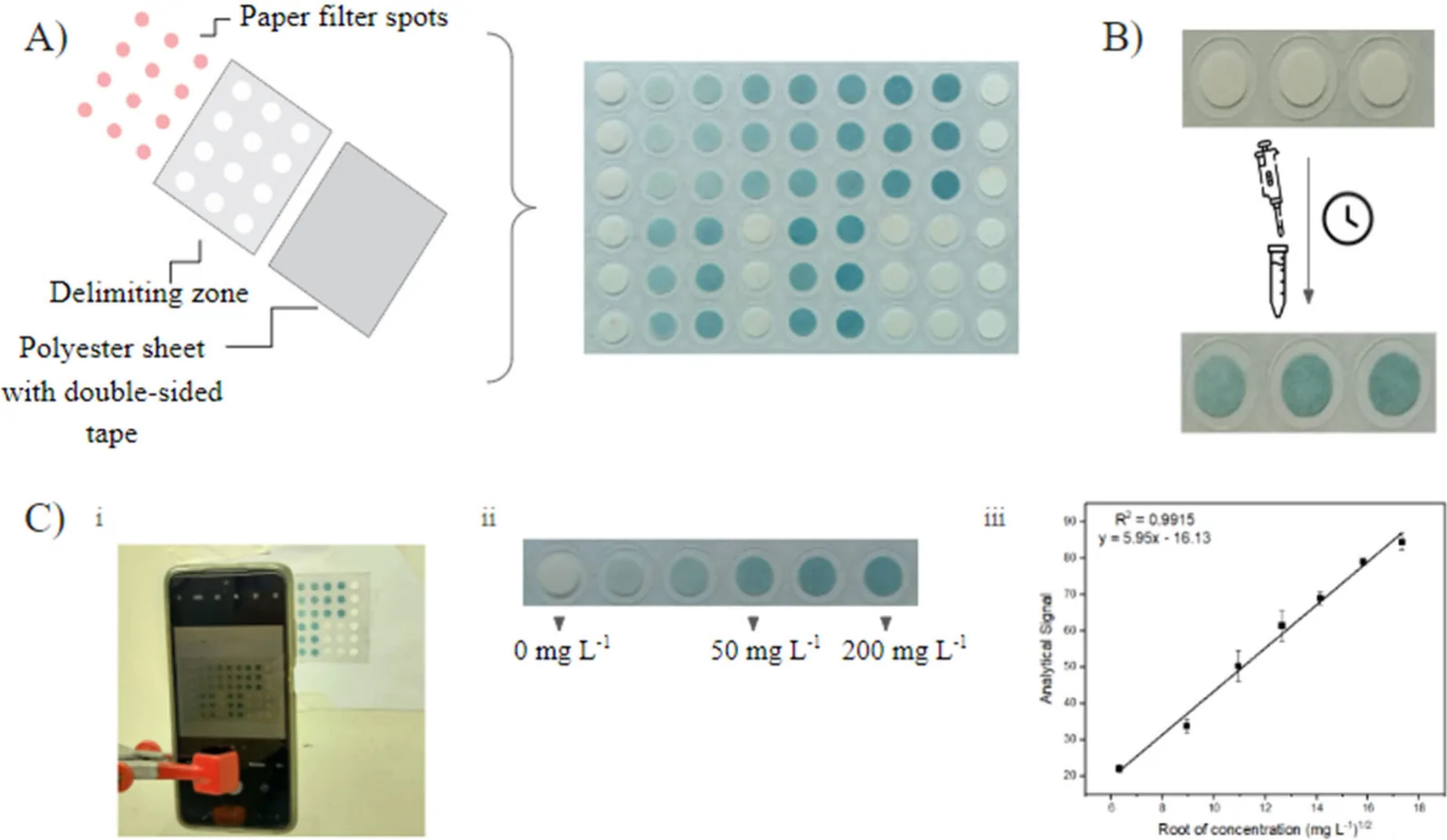
A) Design of the microfluidic paper sensor for quantifying melatonin in pharmaceutical formulations. The assembly of all parts is made with double-sided tape. B) Visible change of color in the spots due to the reaction between Van Urk reagent and melatonin. A small volume of reagent and sample is directly immobilized in the spot using micropipettes. A stable blue color is formed after the necessary time for drying the reagent and the reaction to take place. C) *i*) Using a regular cellphone, it is possible to obtain a picture of the sensor that can be analyzed using the RGB color space in software such as ImageJ. *ii*) Greater melatonin concentrations lead to a more intense blue color through the spot, which can be correlated to the R channel value in the RGB color space. *iii*) An analytical curve is constructed with the colorimetric channel R in the RGB color space.

### 2.2 Reaction Optimization and Use of the μPAD

The reaction used for the quantification of melatonin involves the Van Urk/Erlich (VUS) reagent, which is commonly applied in the forensics area to detect ergoline, an indole alkaloid that is mainly present in the Lysergic Acid Diethylamide drug, or LSD. The reagent traditionally contains 0.1 mol L^–1^ of p-DMAB (p-dimethylaminobenzaldehyde) and 0.01 mol L^–1^ of FeCl_3_ (iron chloride III) in a 4:1 proportion. We optimized the protocol for the use of the reagent on a paper surface, obtaining the following procedure: in a 2 mL microtube, we weighed 5.72 mg of p-DMAB, 4.33 mg of FeCl_3,_ and added 1.5 mL of ethanol, 167 μL of HCl, and 333 μL H_2_SO_4_, so the finals concentrations are 0.1 mol L^–1^ of p-DMAB, 8 mmol L^–1^ of FeCl_3_, 1.0 mol L^–1^ of HCl and 3.0 mol L^–1^ of H_2_SO_4_.

The protocol for using the μPAD is as follows: 8 μL of the reagent was applied directly to the spot, and 40 min were waited for drying, ensuring the complete homogeneity of the reagent. Then, 5 μL of sample/standard solution was applied to the spots, with a 40 min wait for data acquisition while the devices were kept in a closed container containing silica gel, a drying agent. After these steps, the VUS reagent and melatonin react to form a stable blue color (Figure 1B). Although the reaction mechanism has not been fully elucidated, it is suggested to involve oxidation and subsequent condensation of the indole ring with the aldehyde reagent, forming a violet-blue chromophore characteristic of indole derivatives.^18^

### 2.3 Standard and Sample Preparation

The standard solutions of melatonin (Sigma-Aldrich, Brazil) were prepared daily in ethanol, within a concentration range of 40–300 mg L^–1^. For solid samples, we acquired melatonin tablets from a local pharmacy brand (São Carlos, São Paulo, Brazil) with a recommended daily dosage of 0.21 mg of melatonin. Ten melatonin tablets were macerated to obtain a representative average mass, and the final concentration of the melatonin sample solution was *ca*. 210 mg L^–1^ (according to the label, diluted in ethanol). The solubilization of the components was achieved using a vortex mixer for 60 s and an ultrasound bath for 5 min (Cole-Parmer, model 8891). The last step involved filtering the liquid content using a syringe filter (Sartorius, 0.45 μm pore size) and diluting it with ultrapure water to achieve a melatonin concentration of approximately 105 mg L^–1^.

For liquid samples, we used a pycnometer to determine the density of the pharmaceutical solutions. We correlated the sample volume with its mass, avoiding sample loss during the pipetting procedure. After that, we diluted the weighted volume to *ca*.105 mg L^–1^ with ethanol and used a vortex mixer for 60 s to ensure homogenization.

### 2.4 Data Acquisition and Processing

RGB data acquisition was performed using a Xiaomi Redmi 10 PRIME smartphone, equipped with a 50 MP camera (Figure 1C, *i*). After the reaction period was determined (40 min), the sensor was positioned in a specific location in the laboratory, 20 cm from the smartphone, to obtain reproducible images. The analytical signal was defined as the difference between the intensity of the blank solution and the corresponding sample/standard spot, which varies in intensity of the blue color proportionally to the concentration of melatonin (Figure 1C, *ii*). The RGB channel colors were analyzed using ImageJ software (with the plug-in “Readplate”) to obtain the intensity of the R component in RGB color space (Figure 1C, *iii*). An important point regarding the use of the μPAD is that the simultaneous and automated extraction of R channel reflectance values (up to 96 spots) enables high analytical throughput, with an estimated frequency of less than 1 min per analysis, thereby compensating for the time spent on spot drying and analyte interaction. During the drying of the reagent and after adding the sample, the paper platform was stored in a closed environment with silica gel, a desiccant agent, to minimize the influence of humidity present in the spot over the digital image (See Section 2 in the Supporting Information for more details).

### 2.5 High-Performance Liquid Chromatography Assay

The μPAD performance was validated through the comparison with a gold standard technique for pharmaceutical components determination/quality control, HPLC.^19^ We have followed the recommended method for melatonin sample preparation as outlined in the United States Pharmacopeia (USP).^20^

A Shimadzu HPLC (LC-10AD) equipped with a C18 Ascentis Column (15 cm × 4.6 mm × 5 μm) was used, with UV detection (SPD-M202 Diode Array Detector) at 222 nm. For both solid and liquid samples (*n* = 3 for each brand), the chromatographic analysis was performed in the isocratic mode with a mobile phase consisting of monobasic potassium phosphate buffer (0.5 g L^–1^, adjusted to pH 3.5 with phosphoric acid) and acetonitrile (60:40 proportion), the flow rate of 1.5 mL min^–1^ with a total run of 15 min, sample injection volume of 20 μL and PDA detector set at 222 nm. Regarding the preparation of the tablets and liquid samples, the method described in Section 2.3 was applied, with the solvent for the mobile phase changed according to the reference procedure.

## 3 Results and Discussion

### 3.1 Optimization of the Reaction Parameters and Use of the μPAD

#### 3.1.1 Reaction Test

Although the colorimetric reaction using Van Urk’s reagent is well-documented in the literature, its performance in paper-based devices has not been extensively explored. In the first attempt to verify the development of the reaction on the paper surface, we applied 8.0 μL of VUS reagent followed by 2.4 μL of a 210 mg L^–1^ standard melatonin solution. There was a gradual change in the paper color from yellow (the color of the reagent) to green (after mixing the analyte) to blue (the final color from the reaction of the reagent and analyte) (Figure S2B), confirming that even in a different platform, the reaction follows the same behavior.

#### 3.1.2 μPAD Usage Optimization

Determining the volume of each component added to the μPAD is crucial to ensure reproducibility, as the chosen volumes should enable complete color homogenization throughout the spot. To assess this, we performed a kinetic study with different reagent volumes. We followed the change in the R channel for spots with a standard melatonin solution (105 mg L^–1^) and the blank (ethanol) by recording the color transformation in individual spots (*n* = 3) using a smartphone and ImageJ software. Another critical aspect of colorimetric determination is the ideal moment of data acquisition. Every reaction follows a kinetic conversion of reagents into products, and in our scenario, the ideal moment of data acquisition would be when a stable blue color is obtained. We found that the volume of 5 μL produced remarkable color homogeneity throughout the spot, and that the signal change after 40 min was insignificant. Respective Data and more information are given in the Supporting Information (Section S4).

#### 3.1.3 Reagent Optimization

At this stage, the process of melatonin detection using the proposed sensor was optimized, aiming to enhance sensitivity and linearity, while minimizing errors. In addition, it was a particular objective to reduce the acid concentration in the VUS reagent to reduce environmental damage and prevent possible accidents during handling. We have also aimed to use ethanol as the reaction solvent, as it can solubilize the reagents well and is more volatile than water, making the assembly of the μPAD more straightforward and more rapid.

The reagent components were p-DMAB, FeCl_3_, HCl, and H_2_SO_4_. To optimize these, we used a range of concentrations and analyzed how different conditions would affect the parameters of the analytical curve. We can observe that the performance of the optimized reagent (0.1 M p-DMAB, 8 mM FeCl_3_, 2 M HCl, and 3 M H_2_SO_4_) and the original reagent are very similar, which leads us to conclude that the developed method is robust. That is, it can support variations without losing its reliability. For optimal conditions and results for all reagents, refer to the Supporting Information in Section S5.

### 3.2 Validation of the Proposed Sensor

#### 3.2.1 Specificity Test

Specificity refers to the ability of a method to detect a target in the presence of other compounds accurately.^20^ An analytical signal variation range greater than 5% was used to determine if there was interference.^21^ Mannitol, croscarmellose sodium, silicon dioxide, magnesium stearate, menthol, glycerol, and citric acid were tested. None of the solid/liquid medication components significantly interfered with the results obtained. For the complete data, see Section S6.

#### 3.2.2 Analytical Performance of the Proposed Sensor

An analytical curve was constructed with standard melatonin solutions in the range of 40 mg L^–1^ to 300 mg L^–1^ (Figure 2), and a linear response was found using the analytical signal and the square root of the concentration (For more details, see Section S3).^21^

**Figure 2:**
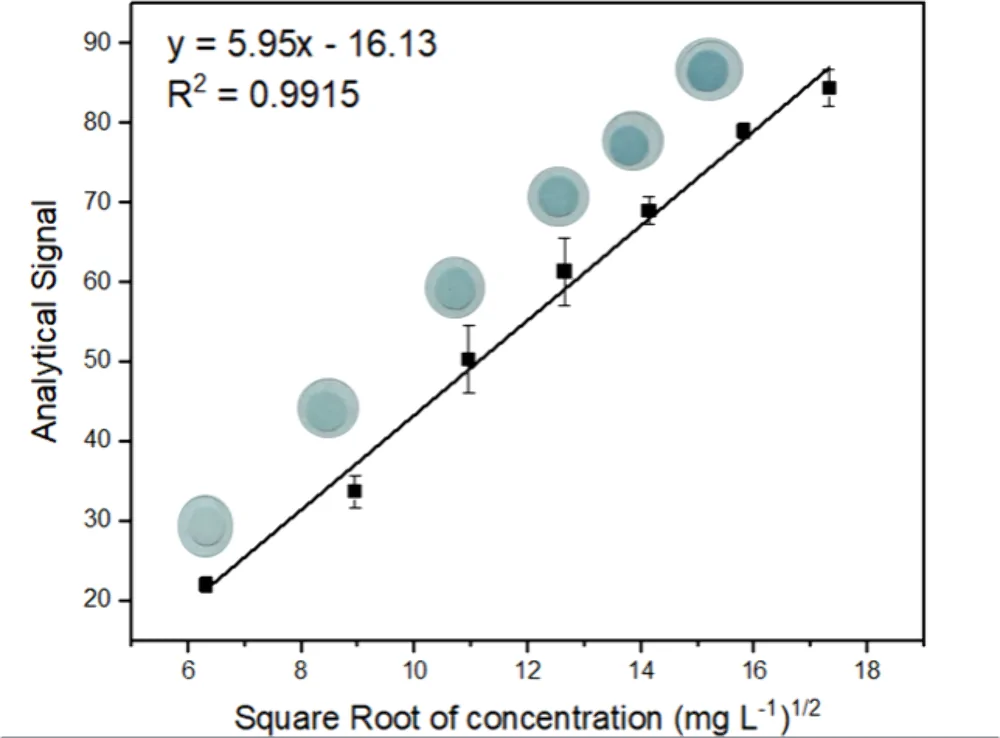
Calibration curve for melatonin standards ranging from 40 mg L^–1^ to 300 mg L^–1^, obtained using the developed analytical method.

The developed method was then applied to the pharmaceutical samples. The precision was evaluated using the standard deviation (SD) for each measurement (*n* = 3), and the results are presented in Table 1. A recovery test was also performed to analyze the impact of the matrix (Table 2), using actual samples fortified with a standard melatonin solution at 50% and 100% of the expected concentration of the actual melatonin sample (105 mg L^–1^).

**Table 1:** Validation of the Proposed Sensor with Real Melatonin Samples from Pharmaceutical Formulations

Form of pharmaceutical formulation	MLT Content (mg ± SD)	Expected MLT content (mg) (label)
Solid	0.22 ± 0.01	0.21
Liquid	0.20 ± 0.05	

**Table 2:** Recovery Test Results Using Fortified Melatonin Solutions

Form of pharmaceutical formulation	MLT initial concentration (mg L^–1^)	Added MLT (mg L^–1^)	Total MLT concentration (mg L^–1^)	Found MLT (mg L^–1^)	Recovery (%) ± SD (%)
Solid	105	52.5	157.5	175	126 ± 0.2
Liquid	105		157.5	150	100 ± 0.5
Solid	105	105	210	239	123 ± 0.3
Liquid	105		210	199	96 ± 0.4

Considering the analyte concentration in the matrix, an acceptable recovery range would be 80–110%.^21^ The analytical performance of the sensor shows excellent precision and accuracy due to the low value of standard deviation (*n* = 3) and the great proximity of results compared to the expected melatonin concentration. However, the recovery test results are not ideal. When redoing the recovery test, a systematic error in the results was observed, leading to two possible hypotheses: inadequate sample preparation (especially for solid samples), and the possibility of parallel reactions due to the reagent’s sensitivity to indole derivatives, which would be consistent with the known behavior of the Van Urk/Ehrlich reaction. The specificity experiments were focused on excipients and vehicles commonly present in the investigated pharmaceutical formulations, since the main objective was to evaluate the applicability of the μPAD for quality control of commercial samples.

#### 3.2.3 Comparison of the Performance of the Proposed Method with the Gold Standard

The last stage of validating an analytical method is to compare it with a previously certified technique or “the gold standard”. In this scenario, an HPLC-certified method was employed to measure the agreement between the results, thereby justifying the reliability of the new method.

An analytical curve was obtained from standard melatonin solutions with a concentration range of 1 to 25 ppm (Figure 3). The certified technique provides a more precise analytical curve than the developed method (Figure 2). However, considering the limitations of a colorimetric technique using digital images, the difference of only 0.7% in R^2^ values indicates that the analytical curve obtained from the developed sensor is also satisfactory.

**Figure 3:**
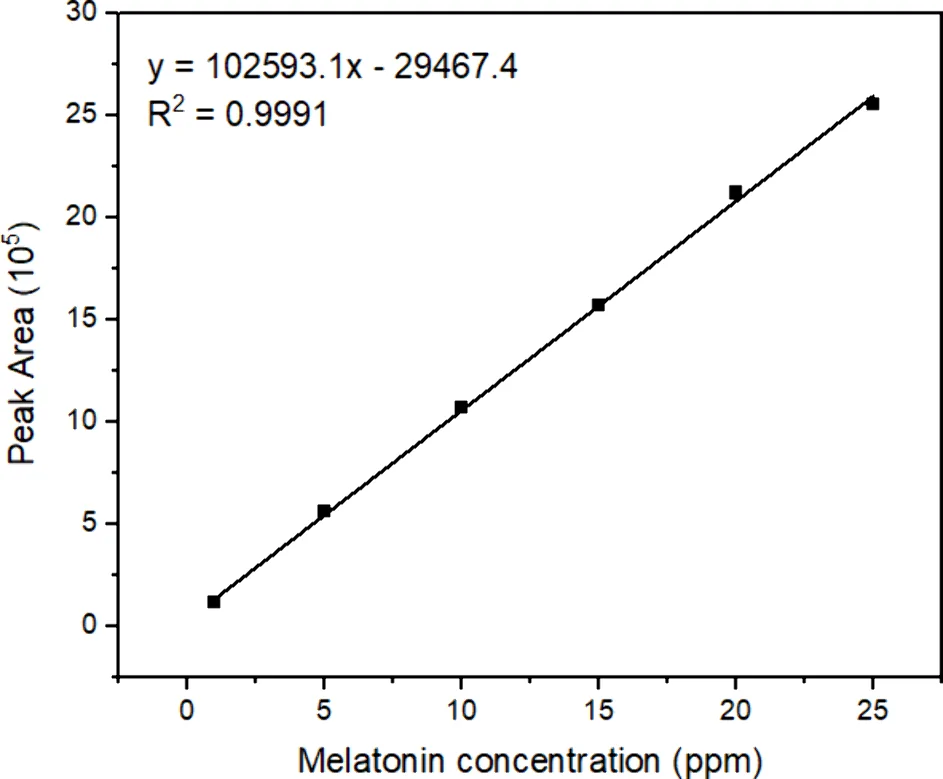
Analytical curve with standard melatonin solutions obtained from the HPLC technique.

Table 3 presents the analytical performance of the sensor and HPLC, while Table 4 displays the results of recovery tests using both methods.

**Table 3:** Comparative Analytical Performance of the Proposed Method and the HPLC Technique for Solid and Liquid Melatonin Pharmaceutical Formulations

Pharmaceutical formulation	Gold standard method (HPLC) (mg/sample) ± SD	Proposed method (mg/sample) ± SD	*t*-test (95% confidence interval)
Solid	0.24 ± 0.03	0.22 ± 0.01	|4.03|
Liquid	0.22 ± 0.01	0.20 ± 0.06	|0.7|

**Table 4:** Recovery Test Results Using Fortified Melatonin Solutions by the Gold Standard and Proposed Methods

Form of pharmaceutical formulation	Gold standard method (HPLC) (mg/sample) ± SD (%)	Proposed method (mg/sample) ± SD (%)
Solid	107 ± 0.2	123 ± 0.3
Liquid	100 ± 0.1	96 ± 0.4

By comparing the analytical performance of the sensor with the HPLC method, different behaviors were observed depending on the sample type. For liquid samples, the calculated *t*-value (−0.70) was lower than the critical value (*t* = 2.776, 95% confidence, *df* = 4), indicating no significant difference between the two methods. In contrast, for solid samples, the calculated *t*-value (−4.03) exceeded the critical value, revealing a statistically significant difference. Thus, while the sensor is suitable for quantifying melatonin in liquid samples, further optimization is needed for solid formulations.

Although there is a significant difference between the linear ranges of the method created for the gold standard, namely 40–300 mg L^–1^ and 1–25 mg L^–1^, its advantages, such as the low cost of the fabrication and use of a microfluidic paper device, the usage of a small volume of reagents, and the good analytical performance, display a robust, specific, and innovative approach to quality control that works for the field of concentrations in food supplements. Considering the Institute’s chemical analysis facility, the average price for using HPLC per sample is $30, while the average cost of the developed sensor is $0.009. For example, the sensor could be used as a screening tool for suspicious samples, allowing pharmaceutical companies to make data-driven decisions.

## 4 Conclusions

We have developed a microfluidic paper-based device for quantifying melatonin in food supplements using a selective reaction based on Van Urk reagent. A colorimetric approach was employed to interpret data obtained from standard cellphone images, based on the “R” parameter of the RGB color space, with optimal reproducibility (standard deviation of 0.01 for solid samples and 0.06 for liquid samples, *n* = 3) and linearity (R^2^ = 0.9915). Comparing the developed sensor with the gold standard method for quantifying hormones, the HPLC technique, it was found that there is no statistically significant difference between the two methods, as indicated by the *t*-test result, which is an outstanding outcome for the future usability of the sensor. We also hope to improve awareness of the quality control of food supplements.

## Supplementary Material



## Notes

Additional information on the design and fabrication of the sensor, signal processing and optimization, and validation steps (PDF)
