# Supplementary material for: μPADs for a Safer Night’s Sleep: An Alternative for Quality Control of Melatonin Pharmaceutical Formulations
Source: ACS Omega. 2026 Aug 14;11(33):49733–8. doi: 10.1021/acsomega.6c03837 (PMC13520511; doi:10.1021/acsomega.6c03837)
Supplement: Supplementary file 1 [file ao6c03837_si_001.pdf]

Supplementary Information

μPADs for a Safer Night's Sleep: An Alternative for Quality Control of Melatonin Pharmaceutical Formulations

Ana Julia Aparecida Ramos[a,b], Laís Canniatti Brazaca[a,b], Eduardo Luiz Rossini[a,b], Manoel de Jesus de Aquino Lima[a,b] and Emanuel Carrilho[a,b,*]

[a] *Instituto de Química de São Carlos, Universidade de São Paulo, 13566-590 São Carlos, SP, Brazil*

[b] *Instituto Nacional de Ciência e Tecnologia de Bioanalítica - Lauro Kubota (INCTBio-LK), 13083-970, Campinas, SP, Brazil.*

S1. Design and fabrication of the sensor

The sensor was created in three steps: 1) Construction of the paper spots, 2) Construction of a delimiting area for the paper spots, and 3) Assembly with a polyester sheet with double-sided tape. We used a CO_2 laser cutter (Laser 600, Combat Laser, Kawai) to cut spots (circles) with diameters of 7 mm (Power: 13, Speed: 80). The spots were cleaned using water three times to remove any possible products of the burn caused by the laser cut and dried in a standard stove at 60 °C. This method quickly produces many spots using a single sheet of paper (on a letter-sized sheet, it is possible to produce more than 1000 spots).

The reaction area around the paper spots is then delimited using a polyester sheet with circular cuts with a slightly bigger diameter (11 mm) to avoid leaks after the impregnation of the reagent and analyte solution. We have also used the laser cutter to cut the polyester sheets (power: 16, speed: 100). The pattern was designed in the laser cutter's software, following the dimensions of 100 mm × 130 mm (length × height), resulting in 48 possible spots.

The paper spots and the area delimiter were assembled on a transparent polyester sheet using double-sided tape. The assembly was then cut using scissors or the laser cutter (power: 16, speed: 100) to secure a cleaner design. A summary of this process can be seen in **Figure S1**.

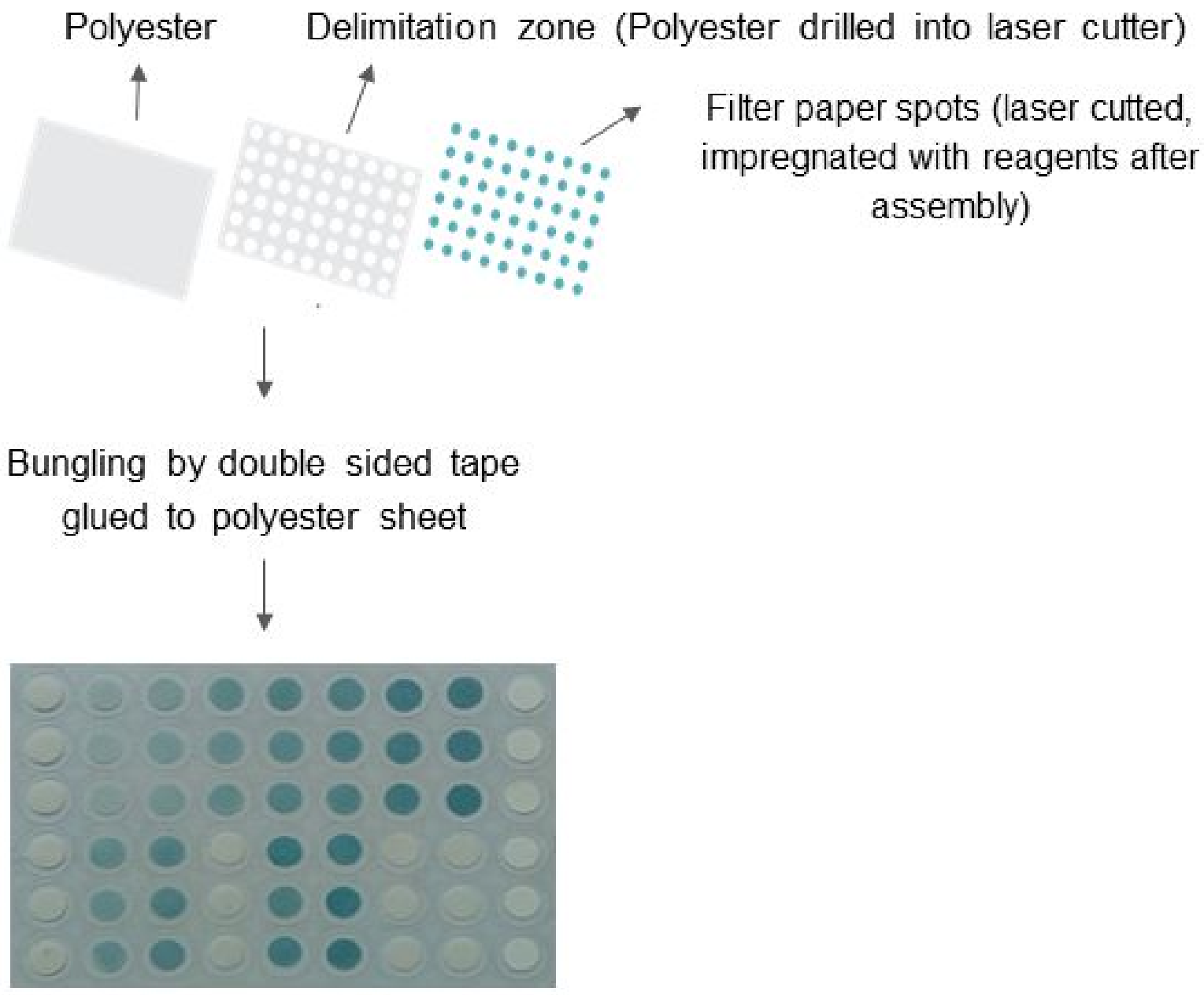

Figure S1. Materials, sensor assembly, and the final device.

S2. Use of a desiccant agent for drying µPADs

One of the disadvantages of the use of µPADs is the low thermal stability of the paper platform. It was noticed that on high air humidity days, 40 min was not enough to promote an adequate drying of the spots, affecting the analytical signal (**Figure S2**). Because of that, during the reagent drying and after adding the sample, the paper platform was stored in a closed environment with silica gel, a desiccant agent.

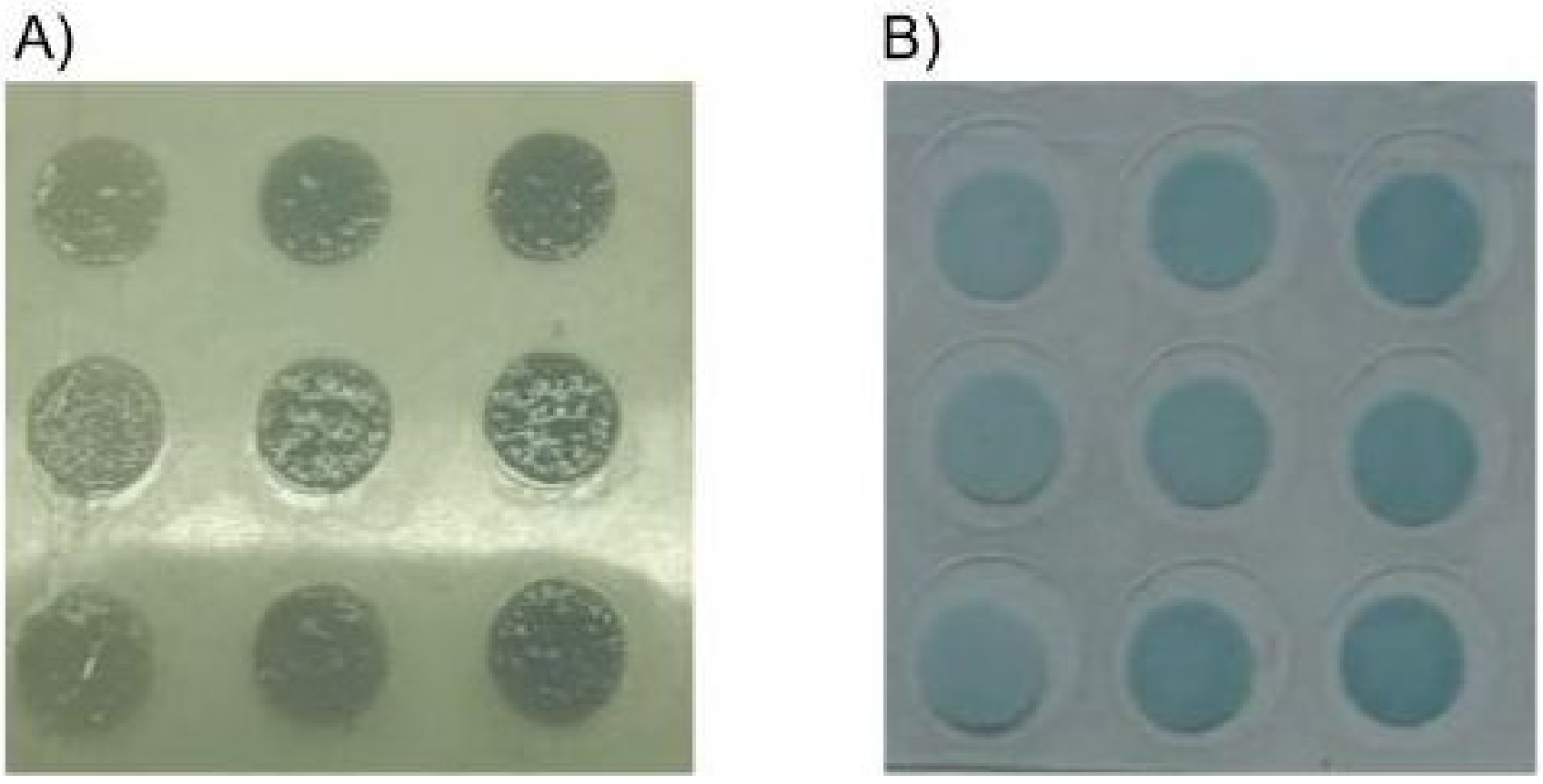

Figure S2. Comparative study between µPADs drying process. A) Outside of a closed environment on a high humidity day and B) Inside a closed environment with a desiccant agent on a high humidity day. It is observable that color perception changes significantly. The pictures were taken after 40 min of the sample immobilization, according to the procedure.

S3. Application of square root of the concentration

When working with colored surfaces on paper, it is known that light is reflected after penetrating its surface, absorbing part of the energy present and going through countless scattering in that process. This phenomenon generates a re-emission ratio (Kubelka Munk ratio) that allows the application of the same logic as for a transmittance analysis for a given concentration range, as long as the parameter of "absolute reflectance" of the surface used is known. In addition to obtaining this type of information being impracticable, it is impossible to ensure the homogeneity of the paper surface nor the total uniformity of color, which generates deviations in a possible linear relationship between concentration and analytical signal. It is a susceptible system; therefore, additional care is needed when acquiring data. Because of this scenario, using relationships such as the one adopted by the square root of concentration is common to obtain the best possible data processing. [1]

S4. µPAD uses optimization

Respective data regarding kinetic analysis to assess the volume of reagent and the ideal moment of data acquisition can be seen in **Figure S3**. We evaluate that using 5 µL was enough to promote color homogeneity without relevant interferences in the drying process in 40 min. The percentage of change in R-channel value after 40 min is 10% to 60 min and 15% to 180 min.

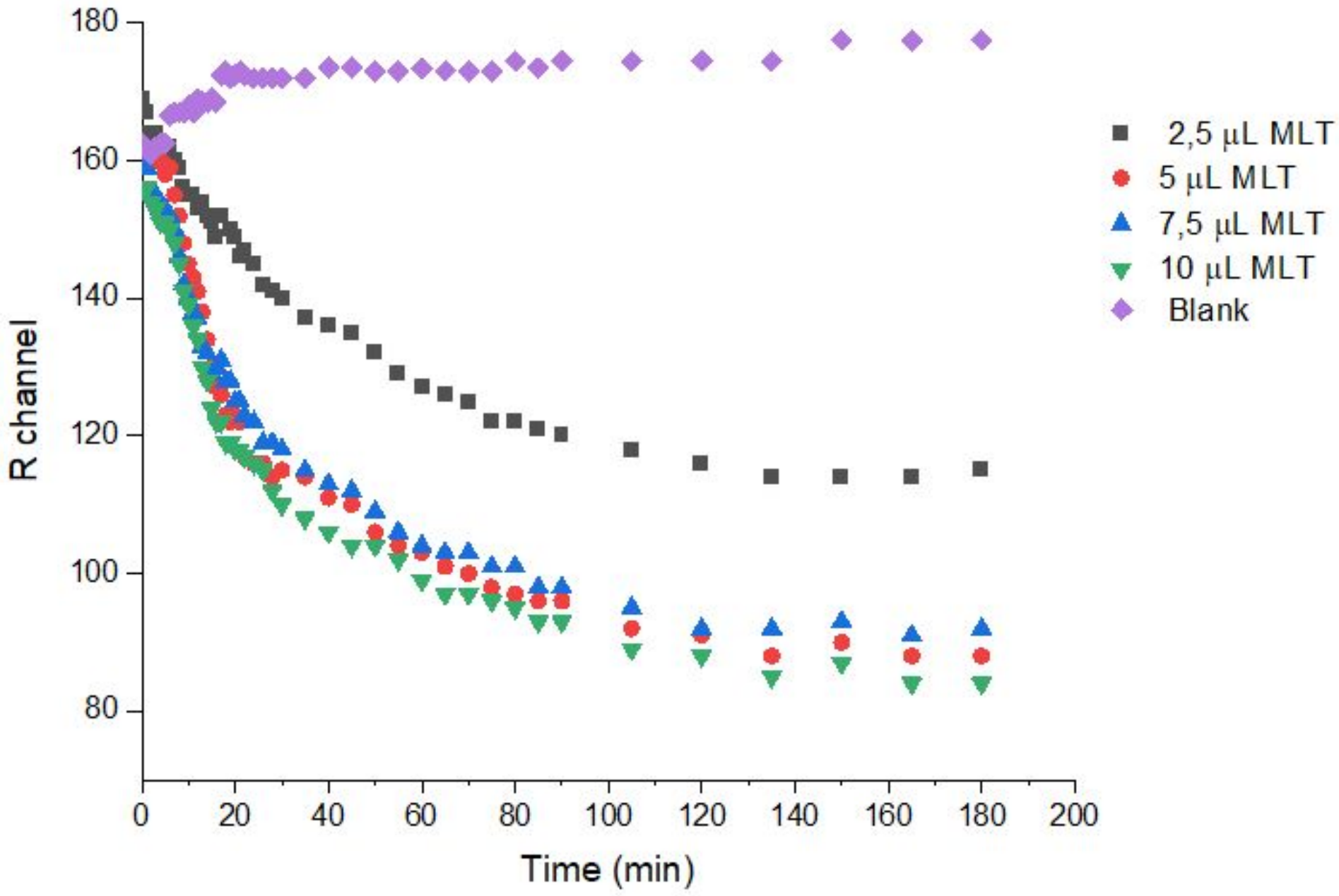

Figure S3. Kinetics of variation of R-channel value with different volumes of melatonin sample (2.5, 5.0, 7.5, 10 µL) and blank solution (ethanol, 5,0 µL). A smaller R channel value indicates an intense blue color, while the blank solution tends to be a white color.

S5. Regent Optimization

The first optimization performed on the reference procedure was a solvent modification. We had water and ethanol as solvents, but to facilitate the optimization of the reagents, it would be interesting to work with only one solvent. Since ethanol is more volatile than water, which is interesting to improve the drying process of the spot, we decided to use only ethanol. We compared the performance of the parameters of interest in analytical curves made with the original reagent and the modified one, seeing a 2% increase in linearity (R^2) but a 20% decrease in sensitivity (**Figure S4**). The absence of one solvent brought benefits to the easiness of procedure execution and provided a more stable system (linearity), which justified the use of ethanol.

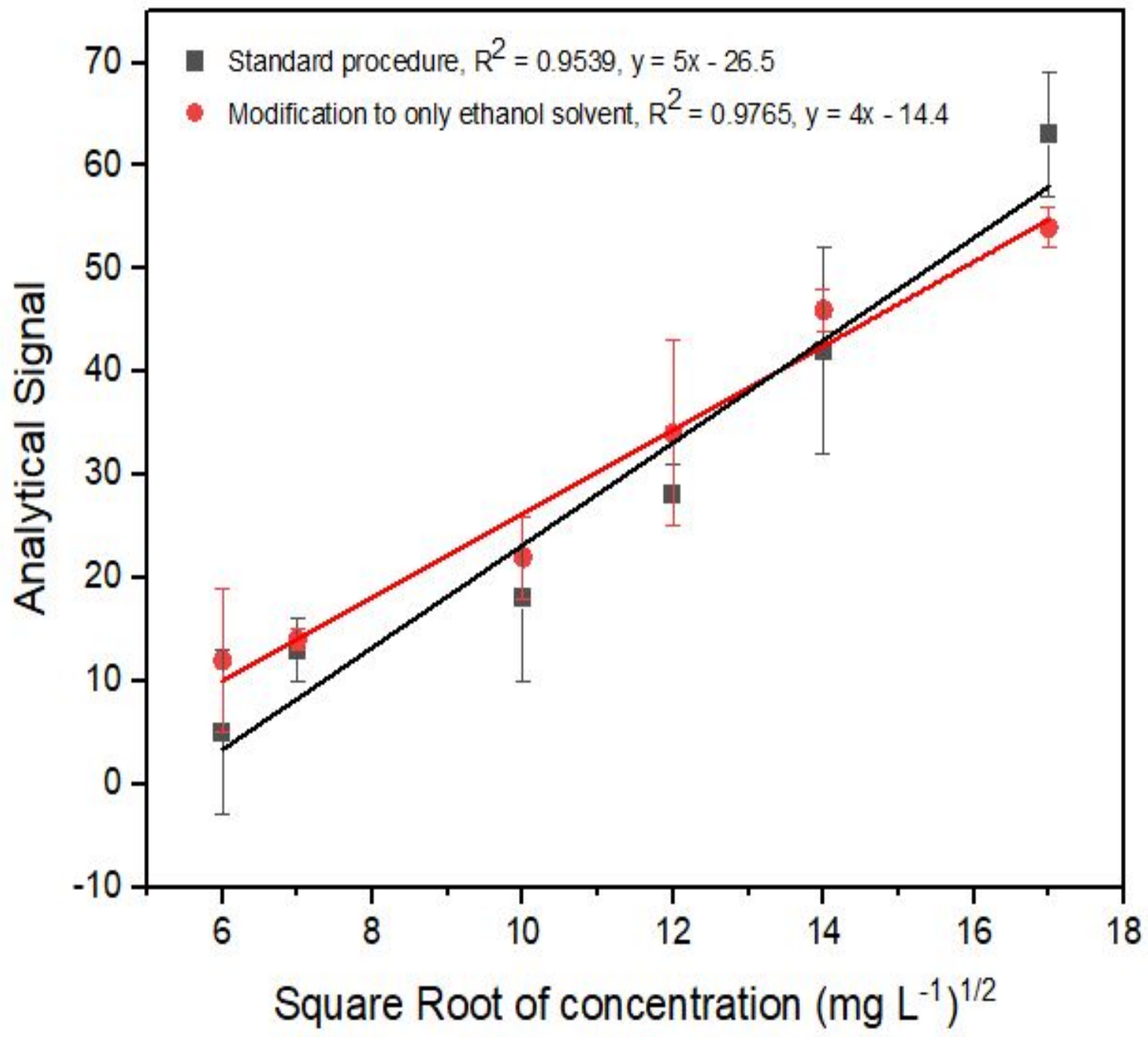

Figure S4. Analytical curve for the first system optimization on the reagent, with the modification for two solvents, water and ethanol, to just ethanol. This modification would accelerate the drying process because of ethanol volatility and facilitate the following optimizations.

Following this, the assessment of reagent concentration was performed. **Table S1** and **Figures S5**, **S6**, and **S7** show the optimal conditions and respective analytical curves. The p-DMAB component followed the same concentration as the reference procedure.

Table 1. Optimal conditions for each component in the reagent, considering the new platform.

Component	Concentration range studied	Optimal condition
p-DMAB	0.02 – 0.14 M	0.1 M, $R^2 = 0.9975$
$FeCl_3$	2 – 10 mM	8 mM, $R^2 = 0.9707$, $y = 4.3x – 16.5$
H_2SO_4	1.0 – 7.0 M	3 M, $R^2 = 0.984$, $y = 5,0x – 9.3$
HCl	0.1 – 2.4 M	1 M, $R^2 = 0.987$, $y = 4.7x – 19.1$

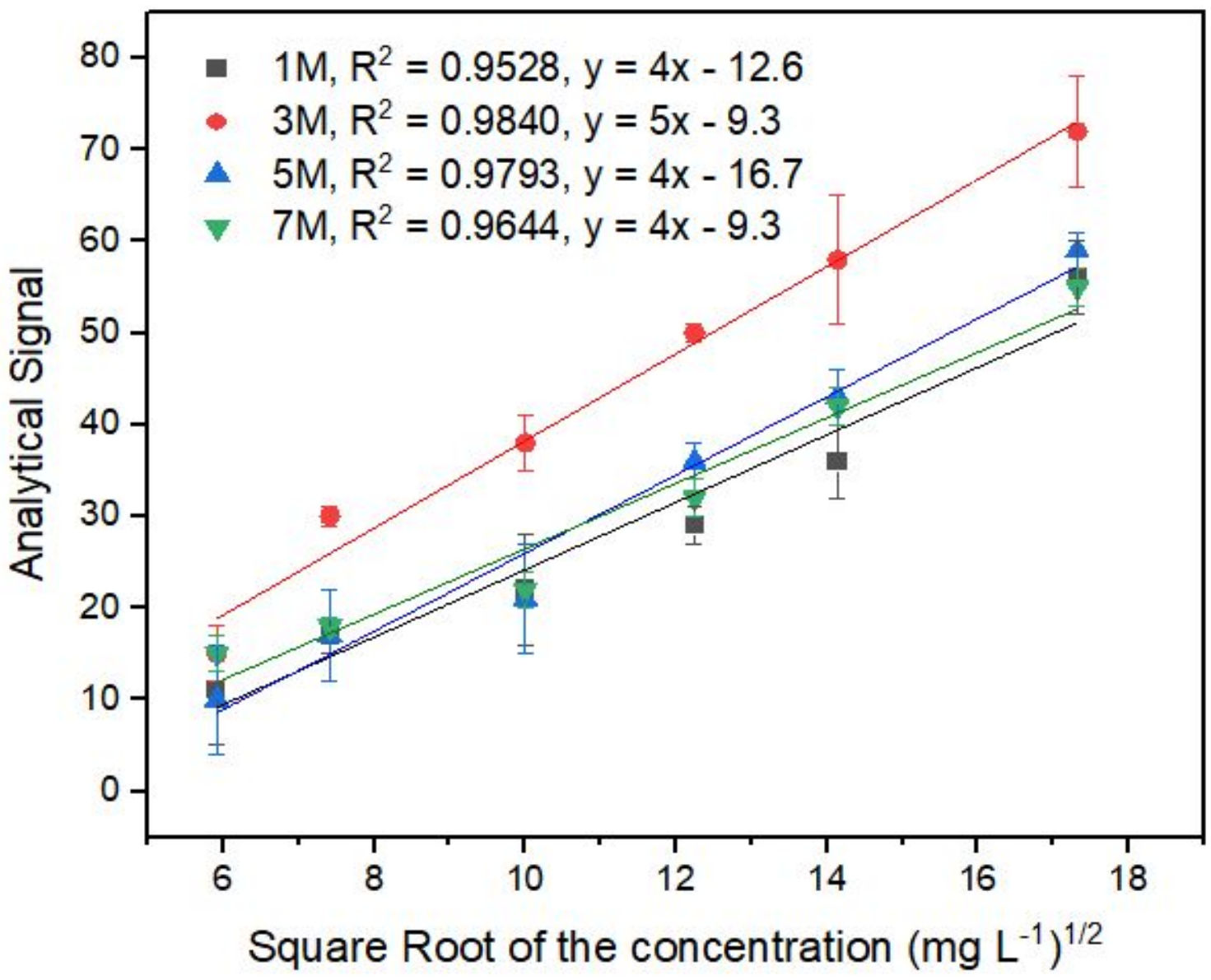

Figure S5. Analytical curves for concentration optimization for H_2SO_4, a component of VUS reagent.

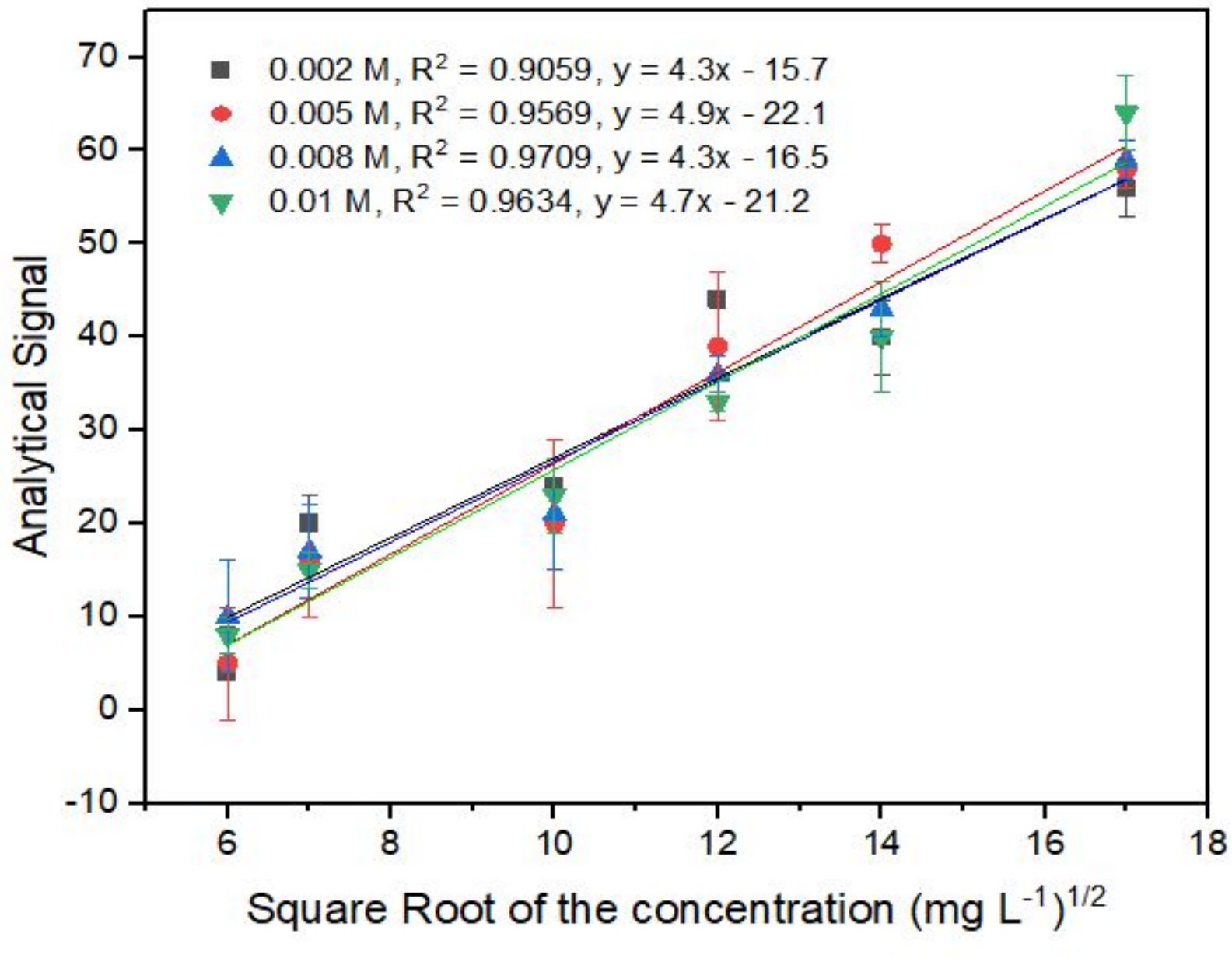

Figure S6. Analytical curves for concentration optimization for $FeCl_3$, a component of VUS reagent.

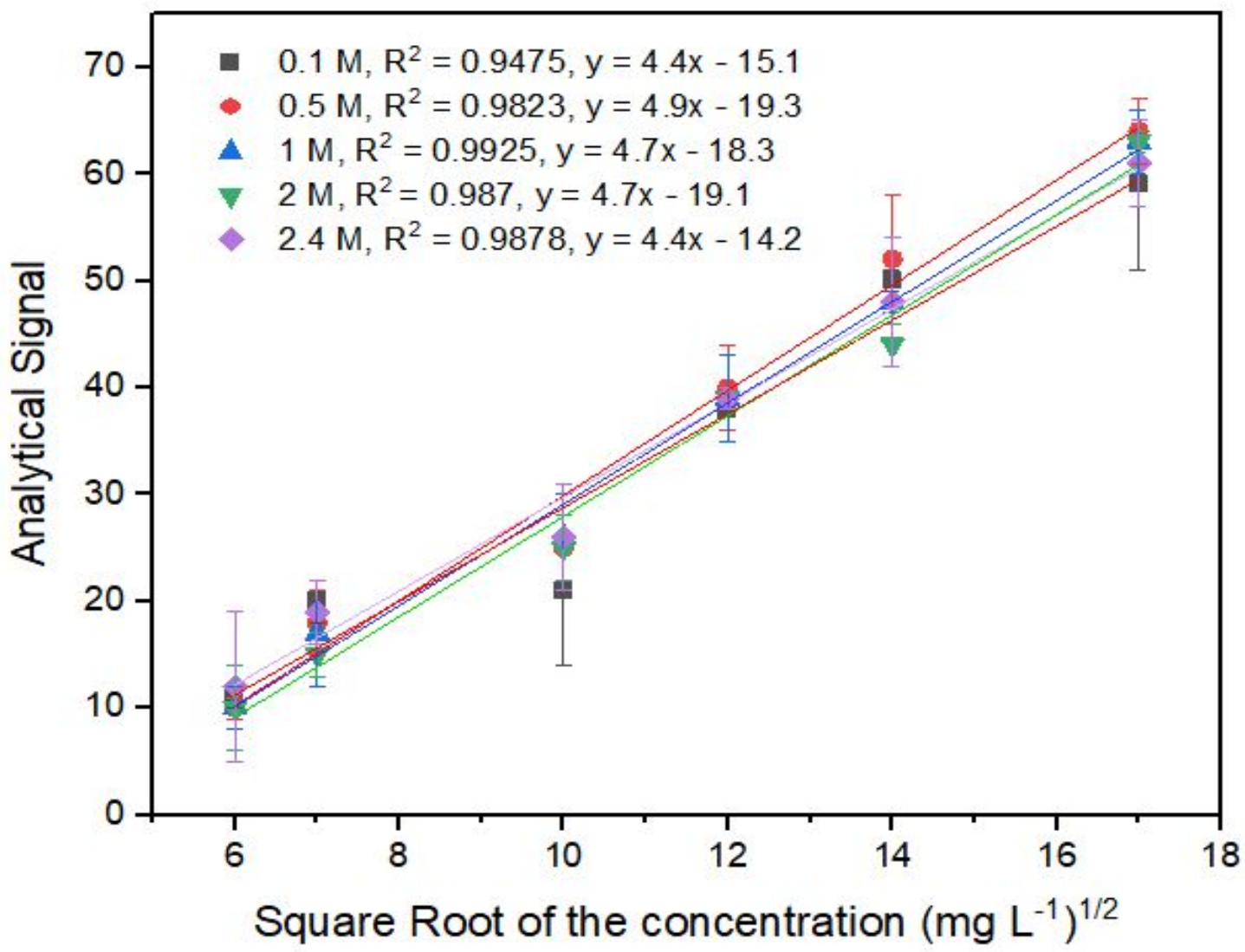

Figure S7. Analytical curves for concentration optimization for HCl, a component of VUS reagent.

S6. Specificity Test

The specificity test was performed based on the possible interaction between the excipients/vehicles and the VUS reagent or between interferents/vehicles fortified with melatonin standard solution and the reagent. These were prepared to mimic the condition of the analyte in the pharmaceutical formulation according to the sample preparation procedure and the concentration range that the sensor could determine. An analytical signal variation range greater than or less than 5% was used to determine if there was interference [2]. Values above 5% indicate that the interferent made the spot bluer and lower, less blue compared to the standard reaction between melatonin and VUS. For both solid and liquid samples, it was possible to see that the excipients/vehicle do not interfere with the analytical signals

Table S2. Specificity test for excipients of solid samples.

Excipients	Concentration (%w/w)	Signal variation based on samples fortified with melatonin 105 mg L^{-1}	Signal variation based on the possible interaction between VUS reagent + excipients
Mannitol	45	−1%	−2%
Croscarmellose sodium	5	−2%	−2%

Silicon dioxide	10	−2%	−5%
Magnesium stearate	10	−2%	1%
Menthol	20	−1%	1%

Table S3. Specificity test for vehicles of liquid samples.

Vehicles	Concentration (%w/w)	Signal variation based on samples fortified with melatonin 105 mg L^{-1}	Signal variation based on the possible interaction between VUS reagent + excipients
Glycerol	90	4%	−2%
Citric acid	5	0%	0%
Menthol	5	3%	4%

References

1. Milani, M. I.; Rossini, E. L.; Castoldi, K.; Pezza, L.; Pezza, H. R. Paper Platform for Reflectometric Determination of Furfural and Hydroxymethylfurfural in Sugarcane Liquor. *Microchemical Journal* **2017**, *133* (2019), 286–292. https://doi.org/10.1016/j.microc.2017.03.046.
2. AOAC International. *Guidelines for Single Laboratory Validation of Chemical Methods for Dietary Supplements and Botanicals*; AOAC International: Gaithersburg, MD, 2016.
